# Does pelvic tilt change with a peri-acetabular osteotomy?

**DOI:** 10.1093/jhps/hnad029

**Published:** 2023-09-09

**Authors:** Jeroen C F Verhaegen, Emin Süha Dedeoğulları, Isabel S Horton, Paul E Beaulé, George Grammatopoulos

**Affiliations:** Department of Orthopaedic Surgery, The Ottawa Hospital - General Campus, 501 Smyth Road, Ottawa, Ontario K1H 8L6, Canada; Department of Orthopaedics, University Hospital Antwerp, Drie Eikenstraat 655, Edegem 2650, Belgium; Orthopedic Center Antwerp, AZ Monica, Stevenslei 20, Deurne 2100, Belgium; Department of Orthopaedics and Traumatology, Hacettepe University, Faculty of Medicine, Sıhhiye, Ankara 06230, Turkey; Department of Orthopaedic Surgery, The Ottawa Hospital - General Campus, 501 Smyth Road, Ottawa, Ontario K1H 8L6, Canada; Department of Orthopaedic Surgery, The Ottawa Hospital - General Campus, 501 Smyth Road, Ottawa, Ontario K1H 8L6, Canada; Department of Orthopaedic Surgery, The Ottawa Hospital - General Campus, 501 Smyth Road, Ottawa, Ontario K1H 8L6, Canada

## Abstract

Change in pelvic tilt (PT) during and after peri-acetabular osteotomy (PAO) is important for surgical planning. The aims of this study were to (i) determine how PT varies throughout the course of treatment in patients undergoing PAO, (ii) test what factors influence the change in PT and (iii) assess whether changes in PT influenced achieved correction. This is an retrospective, single-centre, consecutive case series of 111 patients treated with PAO for global (*n* = 79), posterior (*n* = 49) or anterior dysplasia (*n* = 6) (mean age: 27.3 ± 7.7 years; 85% females). PT was determined on supine, anteroposterior pelvic radiographs pre-, intra-, 1 day, 6 weeks and 1 year post-operatively, using the sacro-femoral-pubic (SFP) angle, a validated, surrogate marker of PT. An optimal acetabular correction was based on the lateral centre-edge angle (25°–40°), acetabular index (−5° to 10°) and cross-over ratio (<20%). There was a significant difference across pre- (70.1° ± 4.8°), 1-day (71.7° ± 4.3°; *P* < 0.001) and early post-operative SFP (70.6° ± 4.7°; *P* = 0.004). The difference in SPF between pre-operative and 1-year post-operative was −0.5° ± 3.1° (*P* = 0.043), with 9% of cases having a difference of >5°. The difference in SFP did not correlate with age, sex, body mass index, type of dysplasia or achievement of optimal acetabular correction (*P* = 0.1–0.9). In the early post-operative period, PT is reduced, leading to a relative appearance of acetabular retroversion, which gradually corrects and is restored by annual follow-up. The degree of change in PT during PAO did not adversely affect fragment orientation. PT does not significantly change in most patients undergoing PAO and therefore does not appear to be a compensatory mechanism.

## INTRODUCTION

Instability secondary to acetabular dysplasia is a common pathology among patients presenting with hip pain [1]. If left untreated, this leads to abnormal loading, increased hip joint contact pressures and early-onset osteoarthritis [2–5]. The degree and extent of acetabular dysplasia can vary greatly, and different patterns of deformity have been described [1]. In patients with minimal degenerative changes and a congruent joint, a re-orientation osteotomy, such as a peri-acetabular osteotomy (PAO), may yield excellent clinical outcomes among patients across the whole spectrum of deformity [6–8]. The outcome following PAO is dependent on the ability to achieve a good correction by improving femoral head coverage for optimum load transfer without introducing impingement [9–12].

**Fig. 1.**
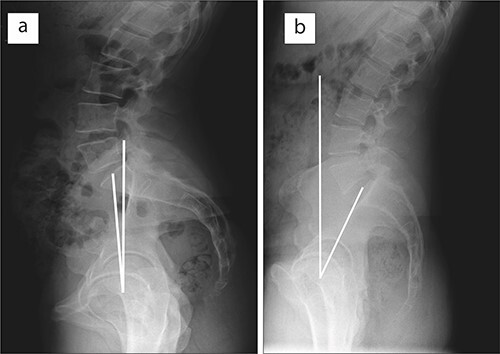
Differences in sagittal PT; a reduced PT leads to anterior rotation of the pelvis in the sagittal plane, decreasing acetabular version (**a**), whereas an increased PT leads to posterior rotation in the sagittal plane, increasing acetabular version (**b**).

Acetabular orientation is directly related to the sagittal position of the pelvis, which is measured by the pelvic tilt (PT) (the angle between the vertical and the line connecting the middle of the sacral S1 plate to the femoral head axis) [13–15]. A reduction in PT leads to the anterior rotation of the pelvis in the sagittal plane, thereby reducing acetabular version (Fig. 1). This is associated with increased anterior and reduced posterior cover of the weight-bearing position of the femoral head [16]. It has been postulated that such compensation manoeuvres take place to alleviate pathomechanics in dysplastic hips [17, 18]. If such compensation manoeuvres are a common occurrence, one would expect for them to be alleviated following the treatment of pathology. However, this has not been shown for either dysplastic or retroverted hips [14, 18, 19].

Whether and how the PT changes during the course of surgical treatment with a PAO (i.e. pre-, intra-, early and long-term post-operatively) are of importance to the surgeon for surgical planning. If PT is significantly different during surgery compared with pre-operatively, then the surgeon might be misled about an inadequate correction. Similarly, if PAO at follow-up is different from pre-operatively, then using the pre-operative tilt as a reference of what orientation to achieve can be deceiving. Thus, the aims of this study were to (i) determine how PT varies throughout the course of treatment in patients undergoing PAO for the treatment of hip instability, (ii) test what factors influence the change in PT and (iii) assess whether changes in PT influenced achieved correction at follow-up. We hypothesize that PT is an independent morphological characteristic, rather than a compensatory one, and therefore will not change significantly following PAO.

## MATERIALS AND METHODS

### Study design

This is a retrospective, consecutive case series from a single, academic centre with a tertiary referral practice for the treatment of young adult hip (YAH) pathology. Following Institutional Review Board approval, the institutional YAH database was queried for PAOs performed between 2011 and November 2021 (ensuring a minimum follow-up of 12 months).

A total of 173 PAOs were identified in 150 patients. The exclusion criteria for participation included significant paediatric pelvic abnormality (*n* = 8), additional femoral procedures at the time of PAO (*n* = 1), incomplete radiographic imaging (*n* = 6), revision PAOs (*n* = 4), cases that remained symptomatic and required further surgery within 2 years post-PAO (*n* = 4) or were converted to Total Hip Arthroplasty (*n* = 3) and lastly, follow-up of less than 3 months (*n* = 15).

### Cohort

The final cohort comprised 134 hips (111 patients) operated by two fellowship-trained surgeons with an interest in YAH pathology. All cases were performed for symptomatic acetabular dysplasia, leading to instability and/or impingement. The mean age of the cohort was 27.3 ± 7.7 years old (range: 16–47), and most were female (*n* = 114, 85%). The mean body mass index (BMI) was 24.7 ± 4.3 kg/m^2^ (range: 18–38).

Pre-PAO morphology was categorized into acetabular dysplasia (*n* = 85) or retroversion (*n* = 40) [2, 20]. Retroversion was defined based on a lateral centre-edge angle (LCEA) of >20° with the presence of cross-over, posterior wall and ischial spine signs [20]. Dysplasia was further subdivided as defined by Wilkin *et al*. into dysplasia secondary to anterior, global or posterior (i.e. retroversion) instability using previously defined thresholds [1]. The most common pre-PAO abnormality was global (*n* = 79), followed by posterior (*n* = 40) and anterior deficiency (*n* = 6). Cases with a lateral CEA of >40° were labelled as pincer femoro-acetabular impingement (FAI) (*n* = 9).

### Surgical technique

PAOs were performed with previously described techniques [21–23]. All were performed on a radiolucent table, with the patient having a general anaesthetic and a paralyzing agent throughout the procedure. Surgery was aided by intra-operative fluoroscopy. Following fixation of the acetabular fragment with 2–4 (4.5 mm) cortical screws, an intra-operative AP pelvis was obtained.

### Radiographic assessments

Supine anteroposterior pelvic radiographs were used for all analyses. Those were obtained in accordance with our institutional protocol which included the following: (i) beam directed perpendicular to the table towards a point midway between the pubic symphysis and the line connecting the anterior superior iliac spines, (ii) a focus distance of 100 cm from the film and (iii) the lower limbs internally rotated 15° [24, 25]. Radiographs were considered adequate if the coccyx was in the same vertical line with the pubic symphysis, with minimal rotation (i.e. iliac wings, obturator foramina and symmetrical radiographic teardrops) [24, 25]. A distance of 1–3 cm from the coccyx to pubic symphysis was not used to evaluate the image quality as this may vary with PT, which was the subject of this investigation, and has been shown to vary beyond these limits in approximately half of the patients with symptomatic acetabular dysplasia despite standardization of an x-ray technique [19].

#### Acetabular parameters

Acetabular measurements were performed on radiographs pre-operatively and at 1-year follow-up, including the following:

- LCEA [26]: an angle between a vertical line passing through the centre of the femoral head and a line passing from the centre of the femoral head to the lateral edge of the bony condensation of the sourcil [27]. An optimal post-PAO correction was considered to have an LCEA between 25° and 40°.
- Acetabular index (AI; Tönnis angle) [28]: an angle between the inter-teardrop line and a line from the medial edge of the sclerotic sourcil to the lateral upturn of the sourcil. An optimal post-PAO correction was considered to have an AI between −5° and +10°.
- Cross-over sign: a sign associated with acetabular retroversion, predisposing to impingement. It has been described to occur when the proximal anterior acetabular rim appears lateral to the posterior rim, creating a ‘figure of eight’ [20, 29, 30].
- Cross-over ratio (COR): an optimal post-PAO correction was considered to have a supine COR of <20% [29, 30].
- Posterior wall sign: a sign of posterior wall deficiency, where the outline of the edge of the posterior wall descends medially to the centre of the femoral head, rather than through the centre point or lateral to it [20].
- Ischial spine sign: a sign considered to be present if the projected triangular shape of the ischial spine protrudes and is visible medially to the pelvic brim.

An optimal acetabular correction had an optimal LCEA, optimal AI and an optimal cross-over ratio.

#### Pelvic tilt

**Fig. 2.**
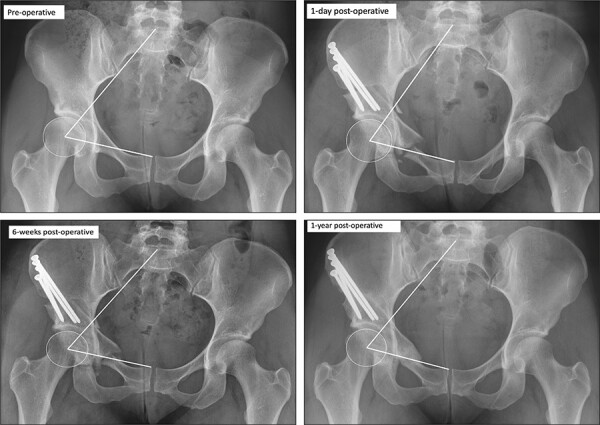
The measurement of the SFP angle at different time intervals during follow-up.

PT was determined from supine, AP pelvic radiographs at various points: pre-operatively, intra-operatively (104/134), at 1 day after PAO, at short-term follow-up (6 weeks) and at 1-year follow-up, and on standing AP pelvic radiographs pre-operatively and at latest follow-up. PT was determined using the sacro-femoral-pubic (SFP) angle, a validated method (Fig. 2) [31, 32]. The SFP is the angle between a line from the midpoint of the S1 superior endplate (found by determining the midpoint of a line between the lateral bodies of L5–S1 facet joints), the centre of one acetabulum and the upper midpoint of the pubic symphysis. Both left and right SFP angles were measured, and where >1° difference was obtained, the mean of the two measurements was used. SFP has been considered a surrogate marker of the true PT, whereby PT equals 75° minus SFP [31, 32]. Thus, as the SFP angle reduces, the PT increases and the acetabulum anteverts. The SFP angle has been shown to be an accurate method to assess the change in PT by subtracting the values obtained in different radiographs, but not of the true value of PT in hip surgery patients, as its accuracy is sensitive to an individual’s pelvic incidence [33]. The difference in the SFP angle (ΔSFP) allowed us to determine the change in PT between various time points. A significant difference in PT was considered when ΔSFP was equal or greater than 5°.

Measurements were performed by an orthopaedic resident (E.S.D.) and repeated by a fellowship-trained hip preservation surgeon (G.G.). Interobserver reliability was calculated using the average correlation coefficient with a two-way mixed model. An intraclass coefficient of 0.811 (95% confidence interval 0.734–0.866) was considered to have excellent reliability (0–1: no–absolute agreement) [14].

### Statistical analysis

Normal distribution of data was tested using Q–Q plots and a Kolmogorov–Smirnov test. Changes in acetabular parameters and SFP were tested for significance using a paired-samples *t*-test. The chi-squared test was used to compare the categorical data. Mann–Whitney *U* tests or Kruskal–Wallis tests were used to compare non-normally distributed variables. Spearman’s rho was used to test for correlations. Variability was defined as 2× standard deviations (SDs). The significance level was set at <0.05. Statistical analysis was performed with SPSS version 27 (IBM Corporation, New York, NY, United States).

## RESULTS

### Change of tilt

**Fig. 3.**
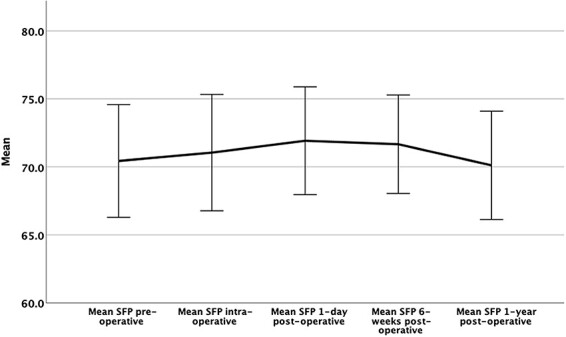
SFB angles at different time points pre-, intra- and post-operatively after PAO.

**Fig. 4.**
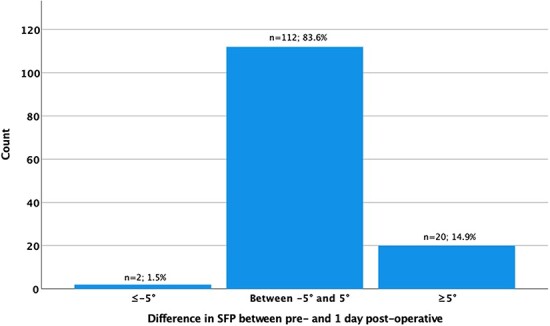
Hips with change in the SFB angle at 1-day post-operative greater than 5° compared with the pre-operative value.

**Fig. 5.**
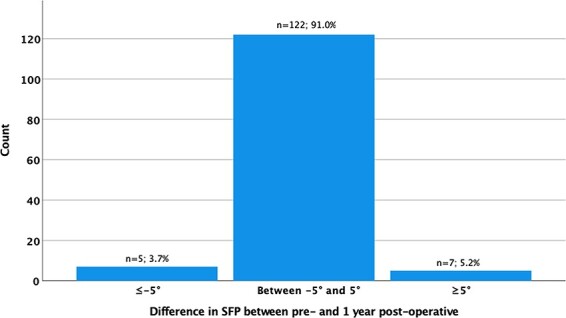
Hips with change in the SFB angle at 1-year post-operative greater than 5° compared with the pre-operative value.

The mean supine SFP and ΔSFP values per time period are detailed in Table I. There was a statistically significant difference between pre-operative (70.1° ± 4.8°), 1-day (71.7° ± 4.3°; *P* < 0.001) and early post-operative SFP values (70.6° ± 4.7°; *P* = 0.004) (Fig. 3). The greatest difference in SFP was between pre-operative and 1-day post-operative (1.7° ± 3.6°). There were 22 cases (18%) with ≥5° difference in SFP values (20 with greater SFP_1-day post_ and two with lower SFP_1-day post)_ between pre-operative and 1-day post-operative (Fig. 4). The difference in SPF between pre-operative and 1-year post-operative was −0.5° ± 3.1° (*P* = 0.043), with only 9% of cases having a difference of ≥5° (five hips with greater SFP_1-year post_ and seven with lower SFP_1-year post_) (Fig. 5).

**Table I.** SFP angles at different time points pre-, intra- and post-operatively after PAO

	*Whole cohort*	*Anterior dysplasia*	*Global dysplasia*	*Posterior dysplasia*	*Pincer-FAI*
Pre-operative SFP (°)	70.1 ± 4.8 (52.9–80.7) (*n* = 134)	61.0 ± 5.7 (52.9–68.9) (*n* = 6)	70.2 ± 4.5 (60.4–80.7) (*n* = 79)	70.9 ± 3.5 (63.5–76.1) (*n* = 40)	71.5 ± 5.5 (60.5–78.3) (*n* = 9)
Intra-operative SFP (°)	71.0 ± 4.7 (57.9–81.4) (*n* = 104)	70.4 ± 7.1 (65.4–75.4) (*n* = 2)	71.1 ± 4.9 (57.9–81.4) (*n* = 62)	70.4 ± 4.3 (60.6–76.3) (*n* = 31)	72.9 ± 4.1 (66.9–78.6) (*n* = 9)
∆SFP pre versus intra (°)	0.5 ± 3.4 (−7.8–10.2)	3.1 ± 4.8 (−0.3–6.5)	0.8 ± 3.2 (−7.8–9.6)	−0.5 ± 3.6 (−7.6–10.1)	1.4 ± 3.7 (−3.0–10.2)
*P*-value^a^	0.068	0.265	0.030*	0.233	0.147
1-day post-operative SFP (°)	71.7 ± 4.3 (59.1–83.5) (*n* = 121)	66.1 ± 2.1 (63.9–69.1) (*n* = 5)	71.8 ± 4.0 (60.5–78.9) (*n* = 71)	71.4 ± 4.5 (59.1–78.9) (*n* = 36)	74.8 ± 4.4 (69.5–83.5) (*n* = 9)
∆SFP pre versus post_1-day_ (°)	1.7 ± 3.6 (−5.9–13.7)	6.0 ± 5.5 (−1.8–13.0)	1.7 ± 2.7 (−2.9–10.3)	0.6 ± 3.9 (−5.9–10.1)	3.3 ± 5.6 (−4.1–13.7)
*P*-value^a^	<0.001*	0.035*	<0.001*	0.183	0.057
6 weeks post-operative SFP (°)	70.6 ± 4.7 (54.6–80.4) (*n* = 104)	61.9 ± 6.7 (54.6–71.4) (*n* = 6)	71.0 ± 4.2 (55.5–79.5) (*n* = 63)	70.9 ± 4.1 (62.3–80.4) (*n* = 29)	72.8 ± 2.1 (69.6–75.9) (*n* = 6)
∆SFP pre versus post_6-weeks_ (°)	0.8 ± 2.9 (−6.8–11.1)	0.8 ± 2.3 (−3.1–3.5)	1.0 ± 2.5 (−6.4–8.5)	−0.1 ± 3.2 (−6.8–6.9)	2.9 ± 4.7 (−1.2–11.1)
*P-value* *^a^*	0.004*	0.211	0.002*	0.416	0.095
1-year post-operative SFP (°)	69.7 ± 5.0 (52.9–82.2) (*n* = 133)	60.6 ± 6.5 (52.9–71.0) (*n* = 6)	69.9 ± 4.7 (58.8–82.2) (*n* = 79)	69.7 ± 4.0 (57.8–75.4) (*n* = 39)	73.5 ± 4.6 (68.3–79.2) (*n* = 9)
∆SFP pre versus post_1-year_ (°)	−0.5 ± 3.1 (−12.5–8.3)	−0.4 ± 3.4 (−5.4–2.1)	−0.3 ± 2.9 (−12.5–6.8)	−1.3 ± 3.4 (−10.9–5.9)	2.0 ± 2.9 (−1.6–8.3)
*P*-value^a^	0.043*	0.392	0.145	0.010*	0.038*

a Paired-samples *t*-test comparing the pre-operative value with intra- and post-operative values.

* Significant if *P* < 0.05.

The mean standing pre-operative SFP was 63.6° ± 3.6°, and the mean post-operative SFP was 62.7°± 5.3° (*P* = 0.079), with a mean difference of −1.3° ± 2.9° at 1-year follow-up (*n* = 12).

### Factors influencing change of tilt

Correlations between ΔSFP and the various factors are detailed in Table II. ΔSFPs did not correlate with age (*P* = 0.110–0.865) and BMI (*P* = 0.067–0.904) and was not different between genders (*P* = 0.091–0.802). ΔSFPs were not different between unilateral and bilateral PAOs (*P* = 0.307–0.729). The type of deformity did influence ΔSFP (*P* = 0.071–0.696).

**Table II.** Correlation between the difference in the SFP angle (∆SFP) and the various factors

	*∆SFP pre versus intra*	*∆SFP pre versus post_1-day_*	*∆SFP pre versus post_6-weeks_*	*∆SFP pre versus post_1-year_*
Age (rho; *P*-value^a^)	−0.079; 0.146	−0.146; 0.110	0.144; 0.145	−0.015; 0.865
BMI (rho; *P*-value^a^)	0.030; 0.800	0.223; 0.067	0.137; 0.310	−0.014; 0.904
Sex (*P*-value^b^)	0.398	0.091	0.479	0.802
Indication (*P*-value^c^)	0.131	0.071	0.310	0.696
Uni- versus bilateral (*P*-value^b^)	0.307	0.729	0.608	0.410

a Spearman’s correlation test.

b Mann–Whitney *U* test.

c Kruskal–Wallis test.

### Acetabular correction and SFP

Acetabular parameters significantly improved with surgery (Table III). AI improved from 10.6° ± 8.7° to 3.5° ± 6.3° (*P* < 0.001). The LCEA improved from 22.7° ± 9.1° to 32.5° ± 7.7° (*P* < 0.001). A cross-over sign was present in 68 hips (50.7%) pre-operatively and in 43 hips (32.1%) post-operatively (*P *< 0.001). The cross-over ratio improved from 17 ± 18% to 7 ± 11% (*P* < 0.001). Acetabular correction satisfying all criteria was seen in 83 (61.9%) of cases. The ability to achieve optimal correction was not different for the three types of dysplasia (*P* = 0.141). There were no differences in any of the ΔSFP measurements between hips with or without optimal correction (*P* = 0.125–0.988).

**Table III.** Acetabular measurements pre-operatively and at 1 year after PAO

	*Pre-operative measurement*	*Measurement at 1*-*year follow-up*	*P-value*
Whole cohort			
LCEA (°) [mean ± SD (range)]	22.7 ± 9.1 (0.0–49.6)	32.5 ± 7.7 (7.2–52.2)	<0.001^a^*
AI (°) [mean ± SD (range)]	10.6 ± 8.7 (−15.5–34.6)	3.5 ± 6.3 (−11.6–23.9)	<0.001^a^*
Cross-over sign (*n*, %)	68 (50.7)	43 (32.1)	<0.001^b^*
COR (°) [mean ± SD (range)]	0.17 ± 0.18 (0.0–0.69)	0.07 ± 0.11 (0.0–0.4)	<0.001^a^*
Posterior wall sign (*n*, %)	44 (32.8)	10 (7.5)	0.427^c^
Ischial spine sign (*n*, %)	40 (29.9)	2 (1.5)	0.509^c^
Anterior dysplasia (*n* = 6)			
LCEA (°) [mean ± SD (range)]	27.1 ± 2.8 (24.3–30.5)	39.5 ± 3.8 (32.3–42.7)	<0.001^a^*
AI (°) [mean ± SD (range)]	9.7 ± 1.8 (7.4–12.6)	1.2 ± 4.0 (−3.1–7.2)	<0.001^a^*
Cross-over sign (*n*, %)	0 (0.0)	0 (0.0)	–
COR (°) [mean ± SD (range)]	–	–	–
Posterior wall sign (*n*, %)	0 (0.0)	0 (0.0)	–
Ischial spine sign (*n*, %)	0 (0.0)	0 (0.0)	–
Global dysplasia (*n* = 79)			
LCEA (°) [mean ± SD (range)]	17.5 ± 6.7 (0.0–29.2)	29.8 ± 7.4 (7.2–51.6)	<0.001^a^*
AI (°) [mean ± SD (range)]	15.3 ± 7.2 (−1.0–34.6)	5.8 ± 5.8 (−9.0–23.9)	<0.001^a^*
Cross-over sign (*n*, %)	25 (31.6)	19 (24.1)	<0.001^b^*
COR (°) [mean ± SD (range)]	0.09 ± 0.14 (0.00–0.44)	0.05 ± 0.10 (0.00–0.39)	0.010^a^*
Posterior wall sign (*n*, %)	8 (10.1)	6 (7.6)	0.485^c^
Ischial spine sign (*n*, %)	3 (3.8)	1 (1.3)	0.962^c^
Posterior dysplasia (*n* = 40)			
LCEA (°) [mean ± SD (range)]	28.1 ± 4.1 (20.5–34.2)	34.1 ± 5.3 (23.5–49.2)	<0.001^a^*
AI (°) [mean ± SD (range)]	5.0 ± 4.1 (−1.5–14.3)	1.6 ± 4.9 (−9.7–9.9)	<0.001^a^*
Cross-over sign (*n*, %)	35 (87.5)	20 (50.0)	0.171^c^
COR (°) [mean ± SD (range)]	0.30 ± 0.14 (0.00–0.59)	0.11 ± 0.13 (0.00–0.40)	<0.001^a^*
Posterior wall sign (*n*, %)	29 (72.5)	3 (7.5)	0.630^c^
Ischial spine sign (*n*, %)	29 (72.5)	1 (2.5)	0.725^c^
Pincer-FAI (*n* = 9)			
LCEA (°) [mean ± SD (range)]	41.7 ± 3.7 (37.9–49.6)	43.9 ± 5.5 (37.0–52.2)	<0.106^a^*
AI (°) [mean ± SD (range)]	−5.1 ± 4.6 (−15.5–−1.1)	−6.3 ± 3.9 (−11.6–−1.1)	<0.482^a^*
Cross-over sign (*n*, %)	8 (88.9)	4 (44.4)	0.556^c^
COR (°) [mean ± SD (range)]	0.39 ± 0.19 (0.00–0.69)	0.09 ± 0.11 (0.00–0.26)	<0.002^a^*
Posterior wall sign (*n*, %)	7 (77.8)	1 (11.1)	0.778^c^
Ischial spine sign (*n*, %)	8 (88.9)	0 (0.0)	–

a Paired-samples *t*-test.

b Chi-squared test.

c Fisher’s exact test.

* Significant if *P* < 0.05.

## DISCUSSION

The effect of PT on acetabular orientation has recently received great attention [34–36]. Studies suggest that patients with dysplasia have increased lumbar lordosis and sacral slope, leading to an increase in anterior coverage [17, 37, 38]. Differences in PT affect joint contact pressure and may thereby influence joint degeneration [39]. There have been limited studies on whether and how PT changes with a PAO and when these changes take place [14, 18, 40]. Furthermore, no studies exist as to what happens intra-operatively and during the early post-operative period, which might influence the assessment of correction. This study illustrates that PT changes minimally between the pre-operative and early follow-up supine positions. This is relevant as surgeons can use the pre-operative radiographs to plan for correction, and what the pelvic position will be at follow-up to judge acetabular orientation. Given that differences in PT are on average small (0.5° ± 3°) between pre-operative assessments and at 1 year post-PAO, compensation manoeuvres are likely to be minimal. The biggest changes identified in PT were intra-operatively and in the early post-operative period. However, the overall amount of the PT change is small. Most often, it leads to an increase in SFP (reduction in PT), resulting in a retroverted appearance of the acetabular fragment. Surgeons should therefore be aware that in the first few weeks post-operatively, the pelvic posture leads to an appearance of inadequate acetabular anteversion, which improves with time. The difference in PT at the time of surgery compared with pre-operatively (based on AP supine radiographs) did not appear to have an influence on the achieved correction. This is most likely because surgeons have taken PT into account during the correction manoeuvre and fixation.

In this study, a change of more than 5° was seen in 9% of cases (increased or decreased PT at follow-up), and a change of 10° was seen in only two patients (both increased) by follow-up. The results of this study are in line with previous studies (Table IV). Roussot *et al*. reported a PT change of greater than 5° in 13% of patients with dysplasia undergoing PAO (all increased; posterior rotation of the pelvis). No patients were observed to have a change in PT >10°[40]. Grammatopoulos *et al*. did not show significant changes in PT in patients with retroversion treated with an anteverting PAO [14]. Similarly, Tani *et al*. demonstrated no difference in the pre-operative and post-operative pelvic sagittal inclination (PSI), nor a change in PSI from supine to standing in patients with acetabular undercoverage undergoing PAO [19]. In contrast to the above-mentioned observations, Daley *et al*. measured the pubic symphysis to sacroiliac (PS-SI) index and reported a significant retro-tilt (>10° in one-third of cohort) at follow-up of 40 patients treated with PAO for bilateral dysplasia [18]. However, the use of the PS-SI index as a measure of PT has not been validated with different pelvic morphologies, and it might be sensitive to individual pelvic morphologies (e.g. pelvic incidence). Daley *et al*. [18] only reviewed bilateral cases, which may have influenced the results; however, in the present study, no differences in the change of PT were seen between uni- and bilateral cases, similar to the findings by Roussot *et al* [40]. All studies mentioned earlier suggest that the observed PT in patients with dysplasia is morphological rather than compensatory, and even if it was compensatory, it does not appear to reverse following PAO. Surgical planning and correction can therefore reliably take place considering the pre-operative, supine, AP pelvic radiograph.

**Table IV.** An overview of literature assessing the change in PT after PAO

*Author*	*Radiographic assessment*	*Acetabular pathology*	*Number included cases*	*Change in PT*
Roussot *et al*. [40]	SFP, PS-SI on supine AP X-ray	Dysplasia	32 bilateral + 32 unilateral PAO	13% PT change >5° using SFP; 10% using PS-SI No PT change >10°
Grammatopoulos *et al*. [14]	PT, PI, SS, APP on pre-operative CT + SFP on supine AP X-ray	Retroversion	6 bilateral + 36 unilateral PAO	No change in PT
Tani *et al*. [19]	PSI on standing and supine AP X-ray	Dysplasia	25 unilateral PAO	No difference in pre- and post-operative PSI
Daley *et al*. [18]	PS-SI index on standing AP X-ray	Dysplasia	40 bilateral PAO	Reduction in anterior PT (30% retro-tilt >10°)

Although changes in PT were minimal, the PT intra-operatively and at early follow-up was different. These differences were small, but a significant variability (2× SD) was observed intra-operatively (6.8°), 1 day post-operatively (7.2°) and at early follow-up (6.2°). In particular, the PT post-operatively was reduced (greater SFP), with a less pelvic retro-tilt, leading to a retroverted appearance of the acetabular fragment. Such postural appearance would be associated with an increase in sacral slope to accommodate for the reduction in PT (keeping pelvic incidence constant) [41]. An increased sacral slope would lead to an increased lumbar lordosis. It is hypothesized that this is related to the iliopsoas. Such posture reduces the lever arm around the pubic cut and reduces psoas-related pain from the cut surfaces. With time, as union occurs, the psoas can slide more easily over the pubis and PT is restored, improving psoas function and lever arm. Surgeons should be diligent with the assessment of the AP pelvic radiographs [9–12] and should assess the features of changes in PT (obturator foramina, iliac spine sign, inlet/outlet appearance of pelvis, SFP and PS-SI) prior to judging the degree of acetabular correction achieved intra-operatively. Furthermore, radiographic evaluation to assess the correction should be performed beyond the 6-week period to allow for the PT to normalize.

Acetabular fragment correction was achieved in a significant proportion of cases. Sixty-two per cent of acetabulae (83/134) satisfied all criteria. The difference in PT between pre-operative and intra-operative pelvic positions did not bear an effect on achieved fragment orientation. This is likely to have occurred because the surgeon took notice of the PT and dialled the degree of correction accordingly. However, further work is required to assess whether navigation software, which considers intra-operative PT, will improve the ability to achieve optimum acetabular fragment orientation.

### Limitations

This study has limitations. First, this was a retrospective study and thus suffers from limitations associated with such a design. Secondly, although supine pelvic radiographs were performed in accordance with our institutional protocol, they were performed by different technicians. Although radiographs were assessed for adequacy prior to conducting measurements, it may be that the centre of the beam was not always centred at the same level and such malpositioning may lead to an erroneous measurement in tilt. Thirdly, the SFP angle was used as an indirect measure to determine the change in PT. Medialization or lateralization of the acetabular centre of rotation could influence SFP measurement. To counteract such an effect of the PAO, we took the average measure between the two sides. Fourthly, the PT change was assessed with SFP only and not by adding a second assessment such as the PS-SI index [18]. The PS-SI index cannot provide an absolute measure of tilt as it is only a ratio; it has been used to describe the direction of movement only, alike other methods [42]. It is sensitive to the pelvic morphology, which can vary greatly in patients undergoing PAO. On the contrary, the SFP has been validated as a reliable tool to measure the PT change and was thus the modality of choice, based on previous studies [40]. Lastly, most assessments were performed in the supine, functional, position and not the standing, weight-bearing one, which arguably more accurately represents the loading situation for the joint. However, the supine assessment is of significance because it is the gold standard assessment and allows for serial evaluations. Therefore, the number of patients with supine and standing radiographs was low, and further studies are necessary to control for compensatory changes that take place in different subgroups. Furthermore, the radiological description of acetabular dysplasia with parameters such as LCEA [26], Tönnis angle [28] and cross-over sign [20] is based on supine AP pelvic radiographs [25]. Lastly, surgical re-orientation and axial imaging occur in the supine position.

## CONCLUSION

Supine PT does not significantly change in most patients undergoing PAO. Therefore, this does not appear to be a compensatory mechanism, but morphological in nature, and in addition provides confidence to the surgeon that the target for correction remains constant during the pre-operative evaluation. In the early post-operative period, PT is reduced, leading to a relative appearance of acetabular retroversion, which gradually corrects and is restored by annual follow-up. The degree of change in intra-operative PT did not adversely affect fragment orientation, likely due to surgeons identifying and correcting for it at the time of surgery.

## Data Availability

The data that support the findings of this study are available from the corresponding author (G.G.) upon reasonable request.

## LEVEL OF EVIDENCE

Level IV, case series.
